# Cryotherapy Reduces Inflammatory Response Without Altering Muscle Regeneration Process and Extracellular Matrix Remodeling of Rat Muscle

**DOI:** 10.1038/srep18525

**Published:** 2016-01-04

**Authors:** Gracielle Vieira Ramos, Clara Maria Pinheiro, Sabrina Peviani Messa, Gabriel Borges Delfino, Rita de Cássia Marqueti, Tania de Fátima Salvini, Joao Luiz Quagliotti Durigan

**Affiliations:** 1 Physical Therapy Division, University of Brasilia, Brasília, Distrito Federal, Brazil; 2 Department of Physical Therapy, Federal University of São Carlos, São Carlos, São Paulo, Brazil

## Abstract

The application of cryotherapy is widely used in sports medicine today. Cooling could minimize secondary hypoxic injury through the reduction of cellular metabolism and injury area. Conflicting results have also suggested cryotherapy could delay and impair the regeneration process. There are no definitive findings about the effects of cryotherapy on the process of muscle regeneration. The aim of the present study was to evaluate the effects of a clinical-like cryotherapy on inflammation, regeneration and extracellular matrix (ECM) remodeling on the Tibialis anterior (TA) muscle of rats 3, 7 and 14 days post-injury. It was observed that the intermittent application of cryotherapy (three 30-minute sessions, every 2 h) in the first 48 h post-injury decreased inflammatory processes (mRNA levels of TNF-α, NF-κB, TGF-β and MMP-9 and macrophage percentage). Cryotherapy did not alter regeneration markers such as injury area, desmin and Myod expression. Despite regulating Collagen I and III and their growth factors, cryotherapy did not alter collagen deposition. In summary, clinical-like cryotherapy reduces the inflammatory process through the decrease of macrophage infiltration and the accumulation of the inflammatory key markers without influencing muscle injury area and ECM remodeling.

Skeletal muscle lesions are responsible for the majority of the functional limitations observed in sports and occupational medicine^1^. After primary injury, muscle regeneration occurs in a highly orchestrated process that involves the activation of muscle satellite cells to proliferate and differentiate into a new muscle fiber^2^ with a constant pattern irrespective of the cause (contusion, strain, or laceration). After muscle injury it is possible to observe four independent phases, despite their etiology: degeneration, inflammation, regeneration, and fibrosis^2^,^3^,^4^. The activation and differentiation of satellite cells is characterized by the rapid upregulation of myogenic differentiation 1 (MyoD) and insulin-like growth factor 1 (IGF-1)^5^,^6^. In addition, *in vitro* and *in vivo* studies indicate that anti-inflammatories such as interleukin-10 (IL-10) and transforming growth factor beta (TGF-β) and pro-inflammatory cytokines such as tumor necrosis factor alpha (TNF-α) and nuclear factor- κB (NF-κB) produced by macrophages could activate satellite cells, stimulating myoblast proliferation and differentiation into myotube formation^7^,^8^.

The fibrosis and remodeling phases of muscle regeneration involve the deposition of Collagen I and III fibers and reorganization of the tissue, which could be induced by TGF-β^9^, IGF-I^10^, and connective tissue growth factor (CTGF)^11^. In addition, matrix metalloproteinases (MMPs) cooperatively degrade all components of the extracellular matrix (ECM)^12^. MMP-2 (or gelatinase A) activity is concurrent with the regeneration of new myofibers probably due to degradation of type IV collagen of the basement membrane during myoblast proliferation, migration, and fusion. MMP-9 (or gelatinase B) activation is related to the early inflammatory phase and to the activation of satellite cells^13^,^14^.

The primary role of cryotherapy post-injury in sports medicine is to reduce pain, swelling, degeneration and inflammation^2^,^15^,^16^,^17^. Cryotherapy is applied in clinical practice with a duration of 20 to 30 minutes, at least 3 times per day in the first 72 hours after soft injury^18^,^19^. Some studies have demonstrated that cryotherapy minimizes secondary hypoxic injury through the reduction of cellular metabolism and injury area^17^,^20^,^21^,^22^,^23^,^24^. Nuno and colleagues^22^ also showed that intermittent cryotherapy reduced injury area approximately 25% compared to untreated rat muscle. Another study demonstrated that cryotherapy for six consecutive hours reduced macrophage infiltration, edema formation, and myonecrosis (desmin ratio) of rat muscle^24^. However, excessive or prolonged cooling would be damaging in clinical practice^25^.

On the other hand, previous studies suggest that cryotherapy, despite decreasing inflammation^24^,^26^, might delay the muscle regeneration process^4^. Cryotherapy could retarded the migration of macrophages in the injured area and the secretion of growth factors such as TGF-β1 and IGF-I expression, which might be harmful for muscle regeneration^4^. They speculated that macrophages play important roles not only for degeneration, but also for muscle regeneration, and therefore the influence of cryotherapy on macrophage activities might be closely related to a delay in muscle regeneration, impairment of muscle regeneration, and redundant collagen synthesis^4^.

Collectively, few studies have evaluated the effects of cryotherapy on muscle regeneration with intermittent and clinical-like protocols related to those used in humans^4^,^22^,^23^. Moreover, no study used cryotherapy for 20 minutes in the first 72 hours after muscle injury, as recommended in clinical practice. Our hypothesis was that clinical-like cryotherapy, applied to the tibialis anterior (TA) muscle immediately after injury, would minimize the inflammatory process, secondary injury area and harmful collagen adaptation. Therefore, the aim of this study was to evaluate the effects of cryotherapy on inflammation (TNF-α, macrophages, NF-κB, TGF-β and MMP-9), regeneration (MyoD, IGF-1 and desmin), the extracellular matrix (Collagen I and III, CTFG, and MMP-2), the injury area and muscle morphology of rat TA post-injury.

## Methods

### Experimental Design

Three-month-old male *Wistar* rats (n = 42; 301.28 ± 17.85 g) were studied. The animals were housed in plastic cages under controlled environmental conditions (12-hour light/dark cycle) with free access to water and standard chow (Socil, São Paulo). The University of São Carlos Ethics Committee approved the experimental procedures (number 059/2010) and the study was conducted in accordance with the National Guide for the Care and Use of Laboratory Animals (National Research Council, 1996). This study was divided into 3 experimental analysis periods: 3, 7 and 14 days after muscle lesion. The rats were randomly distributed into seven experimental groups of 6 animals for each time period: (1) (control group) animals with no interventions; (2) TA muscle injured and analyzed 3 days after injury (L3); (3) TA muscle injured, treated with cryotherapy and analyzed 3 days after injury (L3 + C); (4) TA muscle injured and analyzed 7 days after injury (L7); (5) TA muscle injured, treated with cryotherapy and analyzed 7 daysafter injury (L7 + C); (6) TA muscle injured and analyzed 14 days after injury (L14) and (7) TA muscle injured, treated with cryotherapy and analyzed 14 days after injury (L14 + C).

### Freezing muscle injury

The rats were anesthetized with an intraperitoneal injection of xylazine (12 mg/kg) and ketamine (95 mg/kg) for the surgical procedures. To induce muscle injury in the middle belly of the right TA, the skin around the muscle was trichotomized and cleaned. Then, a transversal skin incision (about 1 cm) over the muscle middle belly was performed, exposing the TA muscle. A rectangular iron bar (40 × 20 mm^2^) was frozen in liquid nitrogen and then kept for 10s on the muscle belly. The same procedure was repeated two consecutive times with a time interval of 30 s. After that, the skin was sutured^27^,^28^. This model induces a homogeny injury area and restricts the surface region of muscle belly^22^,^27^,^29^, which is similar to mechanism of muscle contusion model^2^. Moreover, it is a model of easy applicability that allows a good reproducibility of experiment and less variability in the extension of muscle damage among animals^22^,^27^,^29^.

### Cryotherapy

Under anesthesia, cryotherapy was performed immediately after muscle injury. The animals were maintained in a horizontal position on a plastic table, and the ankle of right hindlimb was maintained by tape for the exposition of the TA muscle skin. The sessions of cryotherapy consisted of the application of a plastic pack filled with crushed ice, maintained by the tape directly on the skin of the right TA muscle^22^,^23^. The first cryotherapy treatment was performed immediately after the TA injury (three sessions of 30 minutes, applied every 2 h). Similar treatment was performed 24 h and 48 h post-lesion.

### Skin Temperature

An infrared thermometer was used to quantify skin temperature over the TA muscle (range: −18 °C and 260 °C; Wurth Temp, Cotia, Brasil). The measurements were performed during cryotherapy treatment and 30 minutes post-cryotherapy.

### Muscle Sample Collection

After the experimental periods, the animals were anesthetized and weighed. Then, the right TA muscles were carefully removed and weighed. The muscles were then divided into two parts at the middle of the belly: the proximal fragment was used for the histological and immunofluorescence analysis and the distal one for the mRNA analysis. For the histological evaluation, the muscle fragment was immediately frozen in isopentane, pre-cooled in liquid nitrogen and stored in a freezer at −80 °C (Forma Scientific, Marietta, Ohio). For the mRNA analysis, the muscle fragment was frozen in liquid nitrogen and stored at −80 °C.

### Muscle injury area

Histological serial muscle cross-sections were obtained (one section of 10 μm for every 100 μm) in a cryostat microtome (Microm HE 505, Jena, Germany) along with the TA middle belly muscles. One image of all muscle cross-sections was acquired at low magnification, and centralized nuclei were counted as a percentage of total muscle fibers. Since the primary injury was standardized for all damaged muscles, possible differences in the final area of injury were considered because of different extensions in the secondary muscle injury. The qualitative analysis of histological sections stained with Toluidine Blue included a description of the stages of tissue repair, involving the presence and type of inflammatory infiltrate, edema, necrosis and immature fibers of all experimental groups^22^,^27^,^30^.

### Immunofluorescence Analysis

The muscle preparation for immunofluorescence assay was previous described^31^. The primary antibody used for immunostaining was: a) rabbit Desmin (1:100 dilution), catalog no. AB-15200 (Abcam, Cambridge, MA), and the secondary antibody was rhodamine red goat anti-rabbit IgG (1:200 dilution), catalog no. Rb394; (Molecular Probes, Eugene, OR); b) rat laminin (1:100 dilution), catalog no. AB-17792 (Abcam, Cambridge, MA), and the secondary antibody was Alexa-Fluor-488- IgG anti-mouse (1:200 dilution), catalog no. A11029 (Molecular Probes, Eugene, OR); c) mouse anti rat CD68 (1:100 dilution), catalog no. MCA341R (ABD Serotec, Kidlington), and the secondary antibody was Alexa-Fluor-488- IgG anti-mouse (1:200 dilution), catalog no. A11029 (Molecular Probes, Eugene, OR); d) rabbit anti-TNFα (1:200 dilution), catalog no. NBP1-19532 (Novus Biologicals, Littleton, CO), and the secondary antibody was rhodamine red goat anti-rabbit IgG (1:200 dilution), catalog no. Rb394 (Molecular Probes, Eugene, OR); e) mouse Anti-Collagen, Type III (1:100 dilution), catalog no. C7805 (Sigma-Aldrich, St. Louis, MO) and the secondary antibody was Alexa-Fluor-488- IgG anti-mouse (1:200 dilution), catalog no. A11029 (Molecular Probes, Eugene, OR); f) mouse Anti-Collagen, Type I (1:100 dilution), catalog no. C2456 (Sigma-Aldrich, St. Louis, MO) and the secondary antibody was Alexa-Fluor-488- IgG anti-mouse (1:200 dilution), catalog no. A11029 (Molecular Probes, Eugene, OR).

For quantitative measurements of immunoreactivity, images of five different regions from the middle-belly of the TA muscles were captured (Axiocam, Carl Zeiss, Jena, Germany) at a final magnification of 20×, with the microscopic setting kept the same for all slides. Regions categorized as degenerative were those which included fibers with hypercontraction, delta lesion, vacuolated and ghost cells, whereas regenerative regions included myoblasts, myotubes and central-nucleate myofibers. In both, the area occupied by fibers positive for the CD68 and TNF-α-labeled antibodies was determined by computer aided image analysis (Image-Pro Express software, Media Cybernetics, Silver Spring, MD, USA) and calculated as percentage. The percentage of CD68 and TNF-α-labeled area (degenerative or regenerative ones) was assessed by multiplying them by 100 and dividing them by the total number of TA muscle fibers. The interstitial space was not considered when the degenerated and regenerated areas were calculated^32^. The ratio of desmin immunostaining cells/total of muscle fibers was used to quantify the area characterized by the presence of regenerating myofibers and calculated as the percentage (Image-Pro Express software, Media Cybernetics, Silver Spring, MD, USA)^33^. Quantification of Collagen I and Collagen III was performed by ImageJ software using the tool color histogram (version 1.41; Wayne Rasband, National Institutes of Health, Bethesda, MA, USA)^31^.

### RNA Isolation and Analysis

RNA was isolated from one frozen fragment of the distal ends of each TA muscle using 1 ml of Trizol reagent (Invitrogen, Carlsbad, CA) according to the manufacturer’s instructions. The extracted RNA was dissolved in hydroxymethyl-aminomethane·hydrochloride (tris-HCl) and ethylenediaminetetracetic acid (TE), pH 7.6, and quantified by spectrophotometry. The purity was assessed by determining the ratio of the absorbance at 260 nm and 280 nm. The integrity of the RNA was confirmed by inspection of ethidium bromide stained 18S and 28S ribosomal RNA under violet ultra-light.Total RNA was reverse transcribed into complementary deoxyribonucleic acid (cDNA) as previous described^31^.

### Oligonucleotide Primers

Primers used for the amplification of products were as follows: GAPDH (forward: CCACCAACTGCTTAGCACC; reverse: GCCAAATTCGTTGTCATACC); RPLP0 (forward: AGGGTCCTGGCTTTGTCTGTGG; reverse: AGCTGCAGGAGCAGCAGTGG) Myo-D (forward: GGAGACATCCTCAAGCGATGC; reverse: AGCACCTGGTAAATCGGATTG); NF-kB (forward: CATTGAGGTGTATTTCACGG; reverse: GGCAAGTGGCCATTGTGTTC); TNF-α (forward: ACCCCCTGAGTCTGCTCAAT; reverse: CCTGGTGGGACTTGGTTGTA); TGF-β (forward: CCCCTGGAAAGGGCTCAACAC; reverse: TCAACCCAGGTCCTTCCTAAAGTC); Collagen I (forward: ATCAGCCCAAACCCCAAGGAGA; reverse: CGCAGGAAGGTCAGCTGGATAG); Collagen III (forward: TGATGGGATCCAATGAGGGAGA; reverse: GAGTCTCATGGCCTTGCGTGTTT); CTGF (forward: CAGGCTGGAGAAGCAGAGTCGT; reverse: CTGGTGCAGCCAGAAAGCTCAA); IGF-1 (forward: GGAGGCTGGAGATGTACTGTGCT; reverse: TGTGTTCTTCAAGTGTACTTCCTTCTG); MMP-2 (forward: CTGGGTTTACCCCCTGATGTCC; reverse: AACCGGGGTCCATTTTCTTCTTT); MMP-9 (forward: GGATGTTTTTGATGCCATTGCTG; reverse: CCACGTGCGGGCAATAAGAAAG); genes.

### Analysis by Quantitative Polymerase Chain Reactions (qPCR)

Detection of mRNA for the different experimental and control samples were performed in a Rotor Gene 3000 (Cobert’s, Sydney, Australia). The amplification mixes contained 1 μl of cDNA sample, 25 μl of SYBR Green fluorescent dye, Master mix (Applied Biosystems, Foster City, CA) and 180 nM of each primer in a final volume of 50μl. Thermal cycling conditions included 10 min at 95 °C, and then 40 cycles every 15s at 94 °C, 30s at 48 °C for MMP-2, MMP-9 and Myo-D, at 56 °C for TNF-α, NF-kB and GAPDH, and at 48 °C for Collagen I, Collagen III, IGF-1, CTGF, TGF-β respectively, and then 1 min at 72 °C, and finally 10 min at 72 °C. For each gene, all samples were amplified simultaneously in duplicate in one assay run. Data were analyzed using the comparative cycle threshold (Ct) method according to the manufacturer’s guidelines (Bulletin No. 2, Applied Biosystems). The GAPDH mRNA was used as internal control^27^,^34^.

### Statistical analysis

The Shapiro-Wilk and Levene’s tests were used to investigate whether the data were normally distributed. As all included variables were normally distributed, a two-way ANOVA (treatment × time interaction) followed by a Tukey HSD post hoc test was performed to compare treatments. Differences were considered significant when p < 0.05. Statistical analysis was performed using the Statistica 7.0 software package (StatSoft Inc., Tulsa, OK, USA).

## Results

### Surface temperature of TA muscle

**Figure 1:**
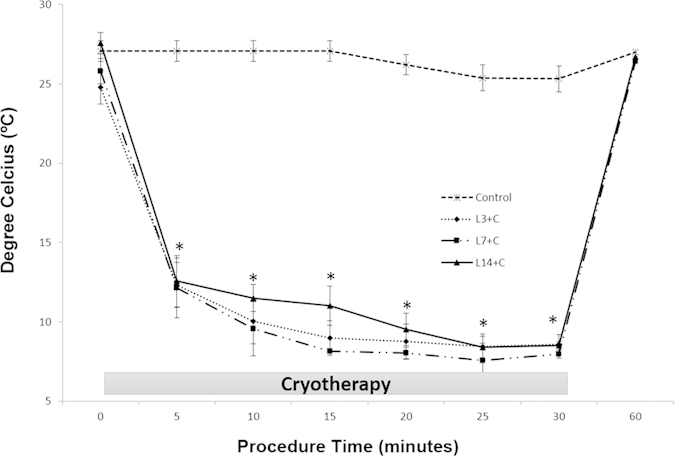
Alterations of the temperatures at the surface of the tibialis anterior (TA) muscle during cryotherapy. Vertical axis shows the temperature in degree Celsius. Horizontal axis shows the time (minutes). Data are expressed as mean ± standard deviation. *p < 0.05: all cryotherapy groups compared to the Control group.

Cryotherapy groups showed a linear decrease in the surface temperature of the TA muscle during its application. Initially, the muscle temperature decreased significantly after 5 minutes of cryotherapy treatment (p < 0.05; Fig. 1). The lowest temperature was observed after 30 minutes of cryotherapy in all different periods when compared to the control group and respective lesion group (L3, L7, L14; p < 0.05). On average, the temperature decreased by 16.19 ± 1.07 °C in the first session, and 17.83 ± 0.89 and 19.06 ± 0.9 °C at 24 h and 48 h, respectively, after 30 minutes of treatment when compared to the initial temperature (p < 0.05; Fig. 1). After cryotherapy sessions, the surface temperature of the TA muscle gradually increased, returning to baseline levels 60 minutes after the first application in all periods. There was no difference in the surface temperature of the TA in the non-treated groups (L3, L7, L14) compared to the control group (p > 0.05). The temperature of the control group decreased 1.83 ± 0.05 °C after 30 minutes of the application and returned to baseline levels after 60 minutes (Fig. 1).

### Animal weight, muscle weight and injury area

There was no statistical difference in animal weight, as well as TA weight in all analyzed groups (p > 0.05; Table 1). The injury area was greatest up to 3 days after the muscle injury compared to the 7 and 14 day groups (p < 0.05; Table 1). Interestingly, cryotherapy did not change muscle injury area among treatment periods (p > 0.05; Table 1).

### Histology of regeneration muscles

**Figure 2:**
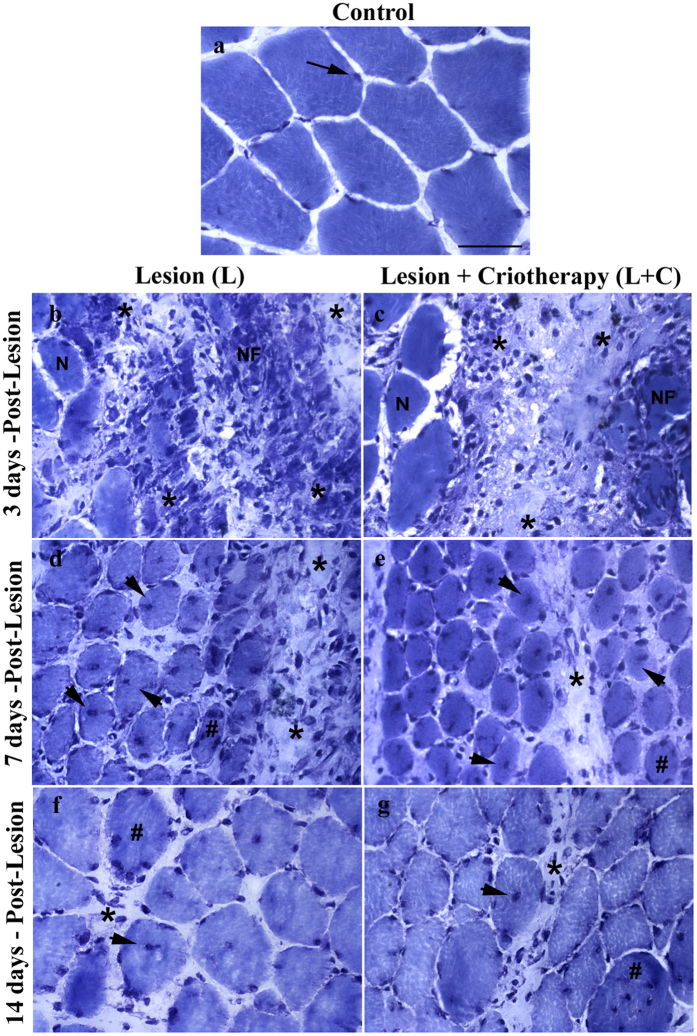
Photomicrographs of cross-section anterior tibialis (TA) muscle of rats evaluated 3, 7 and 14 days (b,d and f) after single injury and evaluated 3, 7 and 14 days (c,e and g) after single injury + criotherapy stained with Toluidine Blue. (**a**) control group without injury. (**b**) signs of muscle tissue damage were identified 3 days after cryolesion by presence of necrotic muscle fibers (NF) and intense presence of cellular infiltration (asterisks). (**d**) note presence of small muscle fibers in intense regeneration process with centralized nucleus (head arrows) and basophilic fibers showing prominent nucleolus (#), also note presence of cellular infiltration (asterisks) 7 days after cryolesion, however with less intensity compared to 3 days. (**f**) muscle fibers in regeneration process 14 days after cryolesion, but still is possible note presence of centralized nuclei fibers (head arrows), basophilic fibers (#) and a minimum presence of cellular infiltration (asterisk) compared with 3 and 7 days after injury. Observe at 3 and 7 days injury + criotherapy groups a evident decrease of cellular infiltration (**c,e**) compared to injury group without treatment by cryotherapy in the same time point (**b,d**). Injury and injury + criotherapy groups were not showed difference with 14 days after cryolesion showing similar regenerative process (**f,g**). (400×). Bar: 50 um.

Cross-sections of TA muscle evaluated 3 days after cryolesion showed several stages of myonecrosis: presence of necrotic muscle fibers, intense presence of cellular infiltration and clear areas among the muscle fibers (Fig. 2b) compared to control muscle fibers (Fig. 2a). As expected, the 7 days-after-injury group showed fewer inflammatory signs, observed by a decrease in cellular infiltration. In this period, it is also possible to note an intense regeneration process through the presence of many small fibers with centralized nuclei, as well as the presence of a large nucleus and prominent nucleolus in basophilic fibers featuring ribosomal activity (Fig. 2d). Regeneration fibers with basophilic and centralized nucleus fibers after 14 days of cryolesion were also noted. In this time, fibers with similar morphology compared to control group were also observed (Fig. 2f,a). Interestingly, cryotherapy had decreased cellular infiltration 3 and 7 days after injury (Fig. 2c,e) compared to injury group at the same time point (Fig. 2b,d). There is no difference in the cellular infiltration of the cryotherapy group after 14 days of muscle injury (Fig. 2f,g). Cryotherapy did not alter morphological aspects of the regeneration process in any evaluated groups (Fig. 2).

### Percentage of muscle fibers with centralized nuclei (%)

Three days after muscle injury, a low number of injured cells with centralized nuclei was observed compared to the control group (p < 0.05; Table 1). However, 7 days after injury this number increased significantly compared to the 3-day period (p < 0.05; Table 1), showing no significant difference compared with cryotherapy treatment (p > 0.05, Table 1). The groups analyzed after 14 days showed the lowest percentage of centralized nuclei (p < 0.05; Table 1) and cryotherapy did not change these results compared with the injured groups (p > 0.05; Table 1; Fig. 2).

### Gene expression by RT-PCR

#### MyoD, IGF-1 and Inflammatory Markers

**Figure 3:**
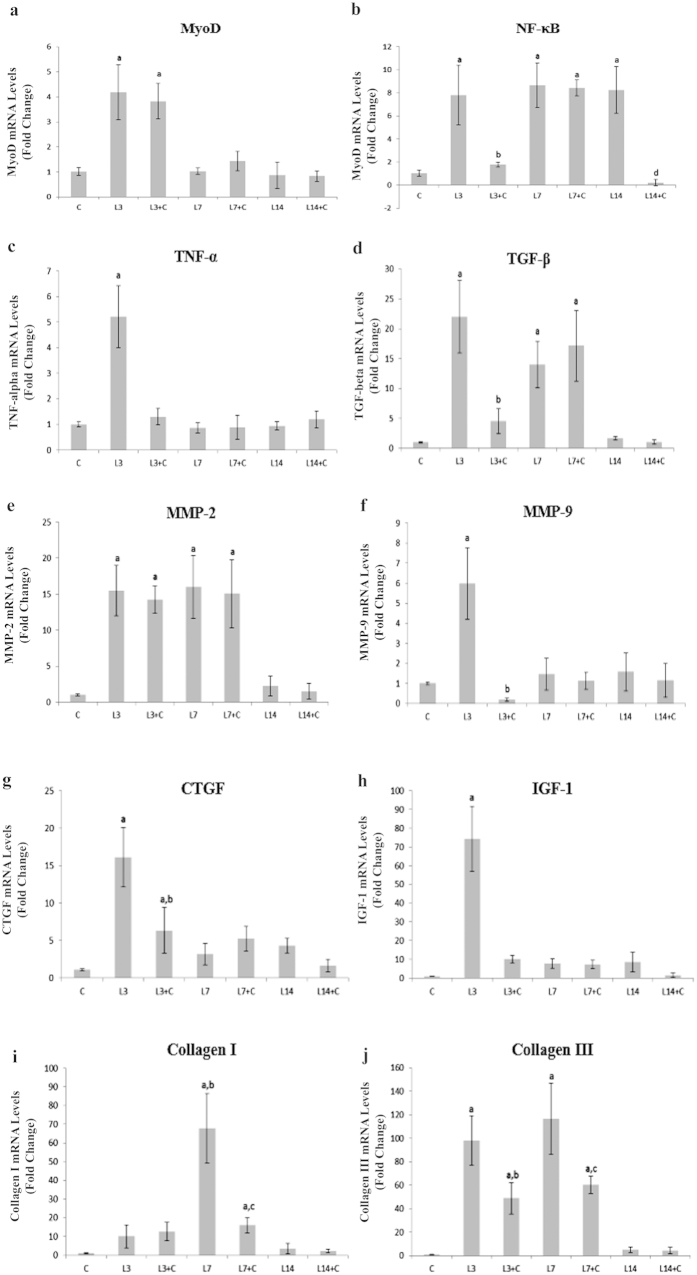
The mRNA levels of MyoD, NF-κB, TNF-α, MMP-9, MMP-2, TGF-β, CTGF, IGF-1, Collagen I and Collagen III of Tibialis anterior (TA) muscle. Data are expressed as mean ± standard deviation. (**a**) = p < 0.05: compared to Control group; b = p < 0.05: compared to L3 group; c = p < 0.05: compared to L7 group; d = p < 0.05: compared to L14 group. Data are expressed as mean ± standard deviation.

Compared to control, the L3 and L3 + C groups showed increased mRNA levels of MyoD (L3: 4.1 fold, L3 + C: 3.8 fold, p < 0.001; Fig. 3a) and returned to baseline values 7 days post-lesion. These data demonstrate cryotherapy did not modify regulation of MyoD observed 3 days after injury. Moreover, cryotherapy groups did not alter MyoD mRNA levels compared to injured muscles in other evaluated periods (p > 0.05; Fig. 3a).

IGF-1 mRNA levels were positively increased only at 3 days post-lesion compared to the control group (L3: 74.4 fold, p < 0.001; Fig. 3h). Interestingly, cryotherapy decreased IGF-1 mRNA levels compared to L3 group (L3 + C: 10.3 fold, p < 0.001; Fig. 3h). However, IGF-1 mRNA levels did not change for other periods of treatment compared with the same period without treatment.

In the muscle injury group NF-κB mRNA levels increased at 3, 7 and 14 days post-lesion compared to control group (L3: 7.8 fold; L7: 8.6 fold and L14: 8.2 fold, p < 0.001; Fig. 3b). Cryotherapy reduced NF-κB mRNA levels at 3 and 14 days compared to L3 and L14, respectively (L3 + C: 6 fold; L14 + C: 8 fold, p < 0.001; Fig. 3b).

The L3 group showed increased mRNA levels of TNF-α (L3: 5.2 fold, p < 0.001; Fig. 3c) compared to the control group and returned to baseline values 7 days post-lesion. L3 + C reduced TNF-α mRNA levels compared to L3 (L3 + C: 3.9 fold, p < 0.001; Fig. 3c).

The muscle injury group increased TGF-β mRNA levels at 3 and 7 days compared to the control group (L3 day: 22 fold; L7 days: 14 fold, p < 0.001; Fig. 3d), and it returned to baseline values 14 days post-lesion. Cryotherapy reduced TGF-β mRNA levels only in the L3 + C group compared to the L3 group (L3 + C: 17.5 fold, p < 0.001; Fig. 3d).

#### Collagen I, Collagen III and MEC transcription factors

Collagen I mRNA levels increased in the L7 group compared to the control group (L7: 67.7 fold, p < 0.001), and returned to baseline values 14 days post-lesion (Fig. 3i). Cryotherapy reduced Collagen I mRNA levels only for L7 + C compared to L7 group (L3 + C: 51.7 fold, p < 0.001; Fig. 3i).

The muscle injury group increased Collagen III mRNA levels at 3 and 7 days compared to the control group (L3: 98 fold; L7: 116.4 fold, p < 0.001; Fig. 3j), and returned to baseline values 14 days post-lesion. Cryotherapy reduced Collagen III mRNA levels at 3 and 7 days post-lesion compared to L3 and L7 groups, respectively (L3 + C: 48.1.5 fold; L7 + C: 55.9 fold, p < 0.001; Fig. 3j).

Only the L3 group increased CTGF mRNA levels compared to the control group (L3: 16.1 fold, p < 0.001), but returned to baseline values 7 days post-lesion (Fig. 3g). Moreover, only the L3 + C group observed a reduction in CTGF mRNA levels, possibly from treatment, compared to the L3 group (L3 + C: 9.8 fold, p < 0.001; Fig. 3g).

MMP-2 mRNA levels increased compared to controls in the L3, L3 + C, L7 and L7 + C groups (L3: 15.4 fold; L3 + C: 14.2 fold; L7: 16 fold; L7 + C: 15 fold, p < 0.001; Fig. 3e) and returned to baseline values 14 days post-lesion. Cryotherapy groups did not alter MMP-2 mRNA levels compared to injury muscles in all evaluated periods Fig. 3e). Muscle injury increased MMP-9 mRNA levels only at 3 days post-lesion compared to control group (L3: 5.9 fold, p < 0.001). The L3 + C group reduced MMP-9 mRNA levels compared to the L3 group (L3 + C: 5.8 fold, p < 0.001; Fig. 3f). It was noted in both 7 and 14 days post-lesion, as well as for cryotherapy in the same periods, that mRNA levels of MMP-9 returned to baseline values, similar to control (Fig. 3j).

### Immunofluorescence

#### Percentage of Desmin Negative muscle fibers (%)

**Figure 4:**
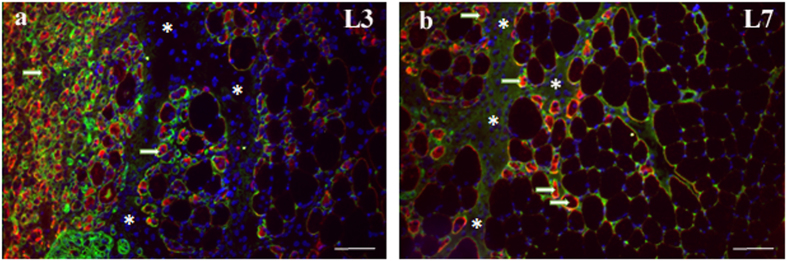
Desmin immunofluorescence in regenerating tibialis anterior (TA) muscles. Frozen muscle sections were immunostained for desmin (red), Laminin (green) and nuclei (blue). Micrographs are representative of muscle cross sections observed on day 3 after muscle damage (L3 group) (**a**) and days 7 after muscle damage (L7 group) (**b**). Serial muscle sections stained for desmin indicate desmin-positive fibers (arrow) and the bundle organization of myofibers was not preserved in injury area (asterisk). Bar, 100 um.

The quantitative analysis of the percentage of desmin negative fibers showed the L3 group had higher values when compared to other groups (p < 0.05; Table 1). It was also possible to note presence of desmin negative fibers in some fibers, possibly in the process of regeneration. (Fig. 4a). L7 and L7 + C groups showed a significant reduction in this percentage when compared to 3-day period (p < 0.05; Table 1), and L14 and L14 + C groups showed lower values when compared to the other groups (p < 0.05; Table 1). There was also no significant difference between cryotherapy and injury groups in all periods (p > 0.05; Table 1). The negative control showed no staining.

#### Percentage of CD68 and TNF-α in muscle fibers (%)

**Figure 5:**
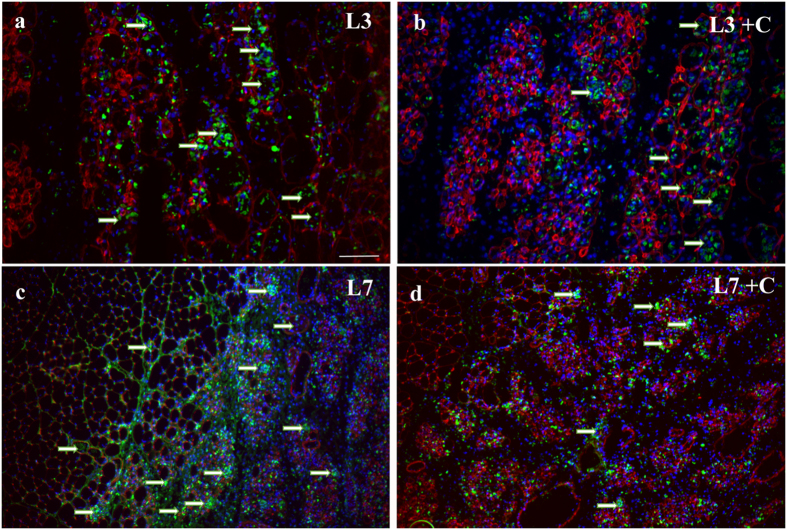
Macrophages (CD68) immunofluorescence in regenerating tibialis anterior (TA) muscles. Frozen muscle sections were immunostained for CD68 (green), desmin (red) and nuclei (blue). Micrographs are representative of muscle cross sections observed on day L3 (**a**), L3 + C (B), L7 (**c**) and L7 + C (**d**). Serial muscle sections stained for CD68 indicate that muscle fibers has been infiltrated by macrophages (CD68 + cells) (arrows). Cryotherapy treatment reduced the percentage of muscle fibers infiltrated by macrophages in 3 and 7 days post-lesion. Bar, 100 um.

The percentage of cells that expressed CD68 significantly increased in the L3 groups when compared to L7 and L14 groups (p < 0.05; Table 1). Interestingly, the percentage of CD68 presented lower values in L3 + C when compared to the L3 group (p < 0.05; Table 1, Fig. 5a/b). Similar results were found in the L7 + C group with the lowest percentage of CD68 compared to the L7 group (p < 0.05; Table 1, Fig. 5c/d). In regards to 14 days after injury, injured muscle presented the lowest value of CD68 and cryotherapy did not affect these levels (p > 0.05, Table 1). The negative control showed no staining.

**Figure 6:**
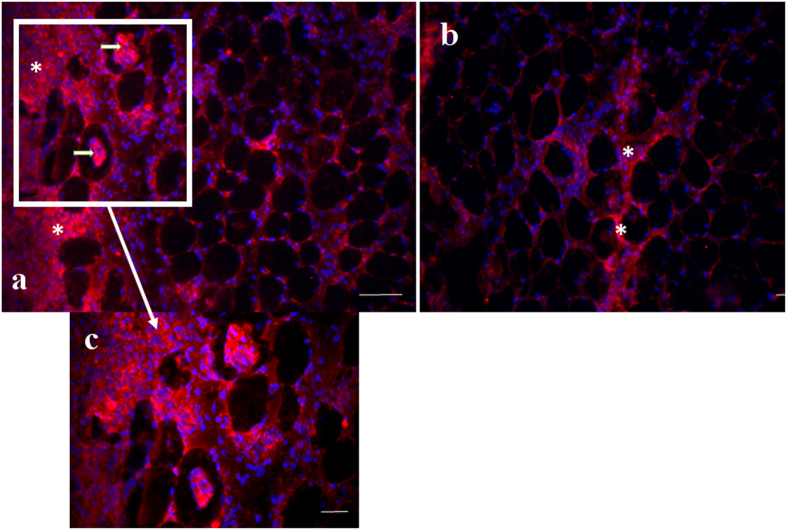
TNF-α immunofluorescence (red) and nuclei (blue) in regenerating tibialis anterior (TA) muscles. Micrographs are representative of muscle cross sections observed on day L3 (**a**) and L3 + C (**b**). C figure are computer-generated merged image (20x of magnification) of the individually captured image of L3 group (**a**). Serial muscle sections stained for TNF-α indicate that muscle fibers has been infiltrated by TNF-α (CD68 + cells) (arrows) and TNF-α are positive stained in injury area (asterisk) Cryotherapy treatment reduced the percentage of muscle fibers infiltrated by TNF-α in 3 days post-lesion. Bar, 100 um.

Regarding the percentage of cells that expressed TNF-α, levels were found to be significantly increased in the L3 compared to L7 and L14 groups (p < 0.05; Table 1). The L3 + C group decreased the expression of TNF-α as compared to the L3 group (p < 0.05; Table 1). It was also noted that cryotherapy prevented TNF-α from infiltrating muscle fibers as well as the injured area (Fig. 6a/b). There were no differences in the percentage of positive TNF-α between L7 and L7 + C groups (p > 0.05; Table 1). In the 14 days after injury group, the lowest values of TNF-α were observed compared to 3 days after injury and there was no difference from the L14 + C group (p < 0.05; Table 1). The negative control showed no staining.

#### Collagen I and III

**Figure 7:**
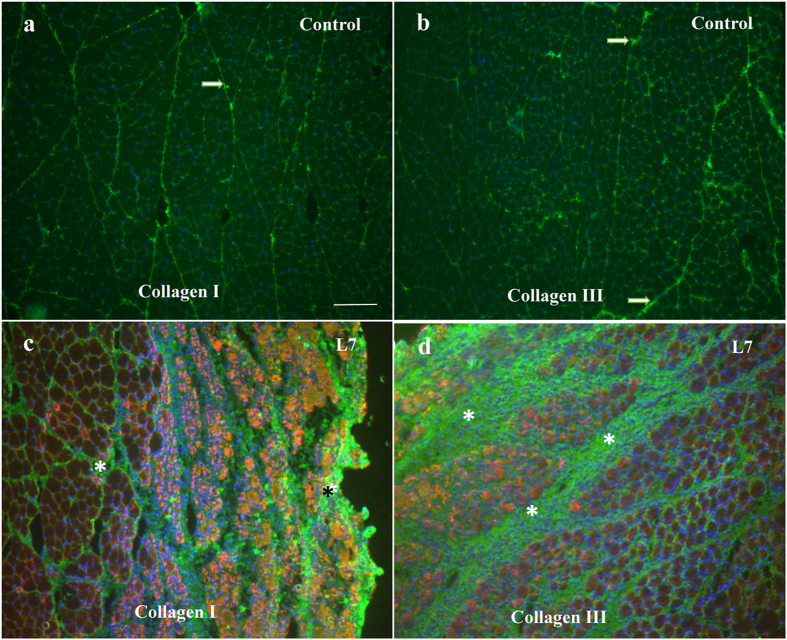
Collagen (green), desmin (red) and nuclei (blue) in regenerating tibialis anterior (TA) muscles. Micrographs are representative of muscle cross sections observed for control group of collagen I (**a**) and collage III (**b**) and for L7 groups collagen I (**c**) and for collagen III (**d**). The muscle fibers expressing collagen I and III were detected in the endomysium and perimysium area of the TA muscle of control group (A and B-arrows). Injury area are positive stained for both collagens 7 days post-lesion (c and d – asterisks). Cryotherapy treatment did not alter the percentage of collagen immunoreactivity fibers in all periods. Bar, 100 um.

Qualitative analysis of the immunofluorescence staining for Collagen I and III showed a positive immunoreactivity in all experimental groups. The muscle fibers expressing Collagens I and III were detected in the endomysium and perimysium area of the TA muscle. Collagen I and Collagen III have increased immunoreactivity only in L7 and L7 + C groups compared to the control (p < 0.05; Table 1; Fig. 7). Compared to Collagen I, it could be seen that Collagen III is more active than Collagen I 7 days after cryolesion (Fig. 7c/d). Cryotherapy treatment did not change these results compared with the injured group. The negative control showed no staining.

## Discussion

These results provide new information about the effects of clinical-like cryotherapy on the molecular pathways involved in TA during muscle. They were characterized by a decreased in inflammatory process, however cryotherapy did not enhance muscle repair and collagen content. The reduction in inflammatory processes could associated to attenuation of pain after muscle injury and could promote structural and functional restoration, which in turn facilitates rehabilitation^35^,^36^. Nevertheless, studies in humans are also necessary to examine this hypothesis, since the physiological significance of this reduction in inflammation, in the face of a lack of effect on repair must be clinically determined.

Although cryotherapy was hailed as advantageous in terms of reducing pain, swelling, degeneration and inflammation post-injury in sports medicine^3^,^15^,^16^,^17^, the results of studies comparing the effectiveness of cryotherapy on muscle regeneration are inconsistent and do not confirm this claim. Schaser *et al*.^24^ found that continuous cryotherapy for six hours applied in closed soft-tissue injury to the left TA compartment attenuated muscle injury and restored functional capillary density associated with markedly reduced intramuscular pressures. Merrick *et al*.^17^ also showed that the application of cryotherapy for five hours after crushing injury reduced the injured area of the sural triceps of rats. Despite the biological contribution from the effects of cryotherapy, those protocols used by Schaser *et al*.^24^ and Merrick *et al*.^17^ are not useful in clinical practice. In addition, continuous cryotherapy lasting for several hours is associated with a certain risk of adverse effects, such as local skin injury^25^,^37^.

Therefore, we only found three studies with results comparable to ours that used intermittent and clinical-like protocols related to those used in humans^4^,^22^,^23^. Oliveira and colleagues^22^,^23^ examined the effect of three sessions of cryotherapy (30 min of ice pack every 2 h), applied after TA muscle injury. The authors concluded that the intermittent sessions of cryotherapy minimized the citrate synthase (responsible for the mitochondrial Krebs’s cycle) and Lactate Dehydrogenase (LDH) activities (terminal enzyme of the anaerobic glycolysis) at 4 h30 and 24 h periods post-lesion, which could be related to the reduction of secondary muscle injury inherent to cryotherapy treatment^4^,^22^,^23^. Data from the present study showed that cryotherapy did not alter the muscle-injured area and the expression of related factors for muscle regeneration (Desmin and MyoD) at 3, 7 and 14 days post-injury. These results are interesting when compared with those of Oliveira and colleagues^22^,^23^. The differences between the present study and Oliveira’s reports are likely related to the cryotherapy protocol and time-point of assessment, since the present study applied cryotherapy in the first 72 hours (3 days of sessions) after lesion, whereas Oliveira evaluated muscle regeneration 4½ hours after muscle injury (one session). The absence of cryotherapeutic effects on muscle injury and markers for muscle regeneration in the present study could be also attributed to the period (3 days, 7 and 14 days after post-lesion) of evaluation in comparison to those studies. Perhaps the period of assessment in Oliveira’s^22^,^23^ studies was not long enough to observe long-term changes in muscle regeneration processes, i.e., they assessed only the positive effects of cryotherapy immediately after muscle lesion.

Interestingly, the negative effects of cryotherapy on muscle regeneration showed by Takagi *et al*.^4^ are related to decreases in resident macrophages in the injury area. Some studies observed that macrophages are crucial in myoblast proliferation and differentiation for forming myotubes^7^,^8^. Satellite cell activity could be also regulated by growth factors and cytokines secreted by neutrophils and macrophages, such as IGF, TNF-α, and TGF-β^6^,^38^,^39^. According to Takagi *et al*.^4^, cryotherapy retarded TGF-β1 and IGF-1 expression secreted by macrophages and impaired muscle regeneration. The present study also observed that cryotherapy decreased TGF-β1 and IGF-1 expression, as well as the percentage of CD68 cells (macrophages) at 3 and 7 days post-injury. In spite of demonstrating that cryotherapy decreased macrophage infiltration in injury area, we did not observe differences in the muscle regeneration process. These results were strengthened by MyoD mRNA levels, which are an important marker of activity of satellite cells^5^,^6^, and they were it was not altered during any time points of cryotherapy treatment.

Surprisingly, the results presented here are in contrast to others^4^,^38^,^39^ in terms of macrophage infiltration being an important regulator of satellite cell activity and muscle regeneration. The complex behavior of satellite cells during skeletal muscle regeneration is tightly regulated through the dynamic interplay between intrinsic and extrinsic factors within satellite cells^6^. Satellite cells are also present in a highly specified niche, which consists of ECM, vascular and neural networks, different types of surrounding cells, and various diffusible molecules. Furthermore, satellite cells, as one of the niche components, also influence each other by means of cell-cell interaction, i.e., integrin cells signaling, and autocrine or paracrine signals^5^,^6^, which was not evaluated in the present study. Despite of being assessed the key factors of satellite cell activation here; we did not exclude the participation of Pax7, Myf5 and Myogenin during muscle regeneration process^5^. Then, it is difficult to infer the spatial and temporal details of satellite cells activity from macrophage infiltration and cytokines signaling patterns due to the regulatory complexity of satellite cells^6^. More studies are necessary to address the possibility of crosstalk of muscle regeneration signaling pathways, such as cDNA arrays and *in vitro* analyses focusing on the interaction between cryotherapy and macrophage modulation involving different myoblast cell populations.

The success of the regenerative processes of myofibers are not only related to the activation of satellite cells, but also the control of collagen deposition in the ECM^40^. The increase of collagen in the ECM could minimize the availability of growth factors and migration of satellite cells, which are required for muscle regeneration^41^. It is well known that exposure to pro-inflammatory cytokines, such as TNF-α, up-regulates TGF-β1, which in turn increases CTGF expression and regulates Collagen I and III turnover^11^,^42^. Our results showed that cryotherapy reduced the expression of type I and III Collagens at 3 and 7 days post-injury, as well as growth factors responsible for their stimulation such as TNF-α, TGF-β1, CTGF and IGF-1 mainly 3 days after lesion. Interestingly, despite cryotherapy decreasing mRNA levels of collagen in the present study, the treatment did not modify the amount of Collagen I and III assessed by immunofluorescence. Taken together, cryotherapy may be a suitable strategy for the recovery of muscle tissue after injury, since the protocol has maintained collagen deposition and ECM remodeling, while reducing inflammation without modifications in muscle regeneration process.

We also showed that muscle lesions increased MMP-2 and MMP-9 mRNA levels. These overall results strongly demonstrate that MMPs up-regulation of mRNA correlates with muscle regeneration, suggesting that ECM remodeling mediated by MMP-2 and MMP-9 is a key process in skeletal muscle fiber degeneration and regeneration. Interestingly, cryotherapy did not alter the MMP-2 expression in agreement with the absence of effects in muscle regeneration, however it was observed a decrease in the MMP-9 expression 3 days post-injury in the cryotherapy group. MMP-9 activity is extensively up-regulated during the first 3 days following cardiotoxic injury in TA muscle, whereas after 3 days following injury, the amount of MMP-9 mRNA and protein begins to decay^14^,^27^, which is in agreement with our results. Previous studies have showed that MMP-9 is secreted by inflammatory cells identified as polymorphonuclear leucocytes and macrophages^14^. According to Kherif^14^, MMP-9 might be associated not only with ECM degradation during inflammation, but also during the initiation of muscle regeneration, probably activating satellite cells^14^.

The decrement in macrophages infiltration could be partially explained by the reduction of MMP-9 expression 3 days post-lesion due cryotherapy^14^. However, it is possible that responsiveness of cytokines by cryotherapy through MMP-9 expression and others inflammatory markers, such as NF-kB and TNF-α, have distinct mechanism, since we did not observe effects on morphology of regenerating muscle as previous described by Takagi^4^. Moreover, it is speculative to mention this relation because satellite cells are modulated by diverse factors^5^,^6^, and we only evaluated the expression of MyoD. Despite of this crosstalk of muscle regeneration signaling pathways, cryotherapy did not alter muscle injury area, desmin protein expression and centrally nucleated (immature) muscle fibers remained at the same level compared to non-treated groups. Curiously, cryotherapy has a different action within the same gelatinase family of MMPs, and this specificity of cryotherapy in altering only MMP-9 expression remains to be elucidated.

Finally, it is important to point that freeze injury model is well recognized to induce necrosis, and subsequently regeneration, in a well-delineated area of skeletal muscles^43^,^44^. Several studies have demonstrated that freeze model induces a homogeny injury area and restrict to surface region of muscle belly^22^,^27^,^28^,^29^. Therefore, cryolesion model cold mimics the mechanism of muscle contusion, since there are superficial and easy applicability that allows a good reproducibility of experiment and less variability in the extension of muscle injury among animals. Despite not having the best model to induce muscle injury, it is possible to consider cryolesion as an excellent method to induce a standardized and clinical-like muscle lesion area, and therefore a useful model to study the effects of treatments in an attempt to recover muscle damage, as cryotherapy^22^,^27^,^28^.

## Conclusion

In summary, clinical-like cryotherapy reduced the inflammatory processes thought to decrease macrophage infiltration and the accumulation of TNF-α, NF-κB, TGF-β and MMP-9 mRNA levels. However, cryotherapy did not change injury area, desmin expression or Collagen I and III protein levels. Our study confirmed the initial hypothesis that cryotherapy could have a beneficial effect on inflammatory process, without affecting the regeneration process after TA injury.

## Additional Information

**How to cite this article**: Vieira Ramos, G. *et al*. Cryotherapy Reduces Inflammatory Response Without Altering Muscle Regeneration Process and Extracellular Matrix Remodeling of Rat Muscle. *Sci. Rep*. **6**, 18525; doi: 10.1038/srep18525 (2016).

## Figures and Tables

**Table 1:** Animals weight, Muscle weight and injury and uninjured cross-sectional area of TA muscle middle belly (mm^2^), desmin negative muscle fibers (%), fibers with centralized nuclei (%), percentage of CD68 and TNF-α immunolabelling per muscle fibers and total area of Collagen I and III immunoreactivity.

Groups	Animal sweight (g)	Muscle weight (mg)	Total area (mm^2^)	Injury area (mm^2^)	Desmin negative (%)	Centralized nuclei (%)	CD 68 (%)	TNF-α (%)	Col I(%)	Col III(%)
Control	295,83 ± 8,56	0,56 ± 0,04	72,79 ± 4,15	—	—	0,07 ± 0,06	—	—	5,34 ± 4,2	12,1 ± 5,7
L3	299,4 ± 11,3	0,57 ± 0,04	68,77 ± 12	29,54 ± 7,94	34,46 ± 5,7	7,29 ± 4,23a	13,21 ± 4,2	31,74 ± 4,4	7,5 ± 5,5	14,1 ± 4,2
L3 + C	273,2 ± 23,36	0,54 ± 0,08	71,70 ± 13,75	28,01 ± 8	35,48 ± 3,2	7,67 ± 3,8a	6,19 ± 0,94b	18,74 ± 2,8b	7,4 ± 3,6	13,5 ± 7
L7	325,6 ± 20,10	0,57 ± 0,07	68,05 ± 11,14	16,12 ± 6,21b	23,02 ± 2,1b	27,1 ± 3,6b	2,13 ± 0,6b	9,89 ± 2,6b	13,1 ± 0,8a,b	22,8 ± 5,6a,b
L7 + C	290,6 ± 37,25	0,53 ± 0,05	65,91 ± 9,48	17,5 ± 6,48c	22,03 ± 2,36c	29 ± 1,6ac	0,30 ± 0,12cd	10,79 ± 1,26c	15,3 ± ,5a,c	24,5 ± 3,9a,c
L14	318 ± 16,04	0,58 ± 0,01	63,83 ± 11,13	4,4 ± 3,47bd	1,26 ± 0,56bd	9,72 ± 1,06abd	0,09 ± 0,02bd	1,05 ± 0,16bd	8,6 ± 2,2	11,5 ± 6,5
L14 + C	309,8 ± 9,23	0,55 ± 0,03	64,54 ± 8,91	4,44 ± 2,53ce	1,71 ± 0,17ce	10,35 ± 2,27ace	0,13 ± 0,06ce	0,85 ± 0,18ce	9,1 ± 3,7	15,3 ± 5,1

Data are expressed as mean ± standard deviation . Normal TA muscle (control); 3 days post-injury(L3); 3 days post-injury treated with cryotherapy (L3 + C); 7 days post-injury (L7); 7 days post-injury treated with cryotherapy (L7 + C); 14 days post-injury (L14); 7 days post-injury treated with cryotherapy (L14 + C).*A = compared to the control group;-B = compared to the L3 group; C = compared to the L3 + C group; D = compared to the L7 group; E = compared to theL7 + C (P < 0,05).

## References

[b1] Rahusen F. T., Weinhold P. S. & Almekinders L. C. Nonsteroidal anti-inflammatory drugs and acetaminophen in the treatment of an acute muscle injury. Am. J. Sports Med. 32, 1856–1859 (2004). 15572312 10.1177/0363546504266069

[b2] Jarvinen T. A., Jarvinen T. L., Kaariainen M., Kalimo H. & Jarvinen M. Muscle injuries: biology and treatment. Am. J. Sports Med. 33, 745–764 (2005). 15851777 10.1177/0363546505274714

[b3] Jarvinen T. A. . Muscle injuries: optimising recovery. Best. Pract. Res. Clin. Rheumatol. 21, 317–331 (2007). 17512485 10.1016/j.berh.2006.12.004

[b4] Takagi R. . Influence of icing on muscle regeneration after crush injury to skeletal muscles in rats. J. Appl. Physiol. 110, 382–388 (2011). 21164157 10.1152/japplphysiol.01187.2010

[b5] Shi X. & Garry D. J. Muscle stem cells in development, regeneration, and disease. Genes Dev. 20, 1692–1708 (2006). 16818602 10.1101/gad.1419406

[b6] Yin H., Price F. & Rudnicki M. A. Satellite cells and the muscle stem cell niche. Physiol. Rev. 93, 23–67 (2013). 23303905 10.1152/physrev.00043.2011 PMC4073943

[b7] Arnold L. . Inflammatory monocytes recruited after skeletal muscle injury switch into antiinflammatory macrophages to support myogenesis. J. Exp. Med. 204, 1057–1069 (2007). 17485518 10.1084/jem.20070075 PMC2118577

[b8] Bencze M. . Proinflammatory macrophages enhance the regenerative capacity of human myoblasts by modifying their kinetics of proliferation and differentiation. Mol. Therapy. 20, 2168–2179 (2012). 10.1038/mt.2012.189 PMC3498804 23070116

[b9] Fu S. C., Wong Y. P., Cheuk Y. C., Lee K. M. & Chan K. M. TGF-beta1 reverses the effects of matrix anchorage on the gene expression of decorin and procollagen type I in tendon fibroblasts. Clin. Orthop. Relat. Res. 431, 226–232 (2005). 15685080 10.1097/01.blo.0000145887.48534.6f

[b10] Doessing S. . Growth hormone stimulates the collagen synthesis in human tendon and skeletal muscle without affecting myofibrillar protein synthesis. J. Physiol. 588, 341–351 (2010). 19933753 10.1113/jphysiol.2009.179325 PMC2821728

[b11] Schild C. & Trueb B. Mechanical stress is required for high-level expression of connective tissue growth factor. Exp. Cell Res. 274, 83–91 (2002). 11855859 10.1006/excr.2001.5458

[b12] Carmeli E., Moas M., Reznick A. Z. & Coleman R. Matrix metalloproteinases and skeletal muscle: a brief review. Muscle Nerve. 29, 191–197 (2004). 14755482 10.1002/mus.10529

[b13] Fukushima K. . Activation and localization of matrix metalloproteinase-2 and -9 in the skeletal muscle of the muscular dystrophy dog (CXMDJ). BMC Musculoskelet. Disord. 8, 54 (2007). 17598883 10.1186/1471-2474-8-54 PMC1929071

[b14] Kherif S. . Expression of matrix metalloproteinases 2 and 9 in regenerating skeletal muscle: a study in experimentally injured and mdx muscles. Dev. Biol. 205, 158–170 (1999). 9882504 10.1006/dbio.1998.9107

[b15] Bleakley C., McDonough S. & MacAuley D. The use of ice in the treatment of acute soft-tissue injury: a systematic review of randomized controlled trials. Am. J. Sports Med. 32, 251–261 (2004). 14754753 10.1177/0363546503260757

[b16] Deal D. N., Tipton J., Rosencrance E., Curl W. W. & Smith T. L. Ice reduces edema. A study of microvascular permeability in rats. J. Bone Joint. Surg. Am. 84-A, 1573–1578 (2002). 12208913

[b17] Merrick M. A., Rankin J. M., Andres F. A. & Hinman C. L. A preliminary examination of cryotherapy and secondary injury in skeletal muscle. Med. Sci. Sports Exerc. 31, 1516–1521 (1999). 10589851 10.1097/00005768-199911000-00004

[b18] Bleakley C. M., McDonough S. M., MacAuley D. C. & Bjordal J. Cryotherapy for acute ankle sprains: a randomised controlled study of two different icing protocols. Br. J. Sports Med. 40, 700–705 (2006). 16611722 10.1136/bjsm.2006.025932 PMC2579462

[b19] Swenson C., Sward L. & Karlsson J. Cryotherapy in sports medicine. Scand. J. Med. Sci. Sports. 6, 193–200 (1996). 8896090 10.1111/j.1600-0838.1996.tb00090.x

[b20] Thorlacius H., Vollmar B., Westermann S., Torkvist L. & Menger M. D. Effects of local cooling on microvascular hemodynamics and leukocyte adhesion in the striated muscle of hamsters. J. Trauma. 45, 715–719 (1998). 9783610 10.1097/00005373-199810000-00016

[b21] Westermann S., Vollmar B., Thorlacius H. & Menger M. D. Surface cooling inhibits tumor necrosis factor-alpha-induced microvascular perfusion failure, leukocyte adhesion, and apoptosis in the striated muscle. Surgery. 126, 881–889 (1999). 10568188

[b22] Oliveira N. M., Rainero E. P. & Salvini T. F. Three intermittent sessions of cryotherapy reduce the secondary muscle injury in skeletal muscle of rat. J. Sports Sci. Med. 5, 228–234 (2006). 24259995 PMC3827564

[b23] Oliveira N. M. L. Durigan J. L., Munin F. S., Schwantes L. B. & Salvini T. F. The effect of intermittent cryotherapy on the activities of citrate synthase and lactate dehydrogenase in regenerating skeletal muscle. Braz. Arch. Biol. Techno. 56, 61–68 (2013).

[b24] Schaser K. D. . Prolonged superficial local cryotherapy attenuates microcirculatory impairment, regional inflammation, and muscle necrosis after closed soft tissue injury in rats. Am. J. Sports Med. 35, 93–102 (2007). 17197574 10.1177/0363546506294569

[b25] Collins N. C. Is ice right? Does cryotherapy improve outcome for acute soft tissue injury? Emerg. Med. J. 25, 65–68 (2008). 18212134 10.1136/emj.2007.051664

[b26] Smith L. L. Acute inflammation: the underlying mechanism in delayed onset muscle soreness? Med. Sci. Sports Exerc. 23, 542–551 (1991). 2072832

[b27] Durigan J. L. . Effects of alternagin-C from Bothrops alternatus on gene expression and activity of metalloproteinases in regenerating skeletal muscle. Toxicon. 52, 687–694 (2008). 18761031 10.1016/j.toxicon.2008.07.018

[b28] Pereira M. G. . Leucine supplementation accelerates connective tissue repair of injured tibialis anterior muscle. Nutrients. 6, 3981–4001 (2014). 25268835 10.3390/nu6103981 PMC4210903

[b29] Pereira M. G. . Leucine supplementation improves skeletal muscle regeneration after cryolesion in rats. PloS One. 9, e85283 (2014). 24416379 10.1371/journal.pone.0085283 PMC3885703

[b30] Salvini T. F., Belluzzo S. S., Selistre de Araujo H. S. & Souza D. H. Regeneration and change of muscle fiber types after injury induced by a hemorrhagic fraction isolated from Agkistrodon contortrix laticinctus venom. Toxicon. 39, 641–649 (2001). 11072042 10.1016/s0041-0101(00)00188-4

[b31] Durigan J. L. . Neuromuscular electrical stimulation induces beneficial adaptations in the extracellular matrix of quadriceps muscle after anterior cruciate ligament transection of rats. Am. J. Phys. Med. Rehabil. 93, 948–961 (2014). 24879548 10.1097/PHM.0000000000000110

[b32] Rocha T. . A. Inflammation and apoptosis induced by mastoparan Polybia-MPII on skeletal muscle. Toxicon. 55, 1213–1221 (2010). 20096299 10.1016/j.toxicon.2009.12.005

[b33] Duguez S., Bihan M. C., Gouttefangeas D., Féasson L. & Freyssenet D. Myogenic and nonmyogenic cells differentially express proteinases, Hsc/Hsp70, and BAG-1 during skeletal muscle regeneration. Am. J. Physiol. Endocrinol. Metab. 285, E206–215 (2003). 12791605 10.1152/ajpendo.00331.2002

[b34] Marqueti R. C. . Gene expression in distinct regions of rat tendons in response to jump training combined with anabolic androgenic steroid administration. Eur. J. Appl. Physiol. 112, 1505–1515 (2012). 21842416 10.1007/s00421-011-2114-x

[b35] Bleakley C. . Cold-water immersion (cryotherapy) for preventing and treating muscle soreness after exercise. Cochrane Database Syst. Rev . 2, CD008262 (2012). 10.1002/14651858.CD008262.pub2 PMC6492480 22336838

[b36] Hubbard T. J., Aronson S. L. & Denegar C. R. Does Cryotherapy Hasten Return to Participation? A Systematic Review. J. Athl. Train. 39, 88–94 (2004). 15085216 PMC385267

[b37] Knobloch K., Spies M., Busch K. H. & Vogt P. M. Comment on report from Schaser and coworkers. Am. J. Sports Med. 35, e1; author reply e2-3 (2007). 17704397 10.1177/0363546507304331

[b38] Grounds M. Towards understanding skeletal muscle regeneration. Pathol. Res. Pract. 187, 1–22 (1991). 2027816 10.1016/S0344-0338(11)81039-3

[b39] Karalaki M., Fili S., Philippou A. & Koutsilieris M. Muscle regeneration: cellular and molecular events. In vivo. 23, 779–796 (2009). 19779115

[b40] Gillies A. R. & Lieber R. L. Structure and function of the skeletal muscle extracellular matrix. Muscle Nerve. 44, 318–331 (2011). 21949456 10.1002/mus.22094 PMC3177172

[b41] Hindi S. M., Shin J., Ogura Y., Li H. & Kumar A. Matrix metalloproteinase-9 inhibition improves proliferation and engraftment of myogenic cells in dystrophic muscle of mdx mice. PloS One. 8, e72121 (2013). 23977226 10.1371/journal.pone.0072121 PMC3744489

[b42] Kahari V. M., Chen Y. Q., Su M. W., Ramirez F. & Uitto J. Tumor necrosis factor-alpha and interferon-gamma suppress the activation of human type I collagen gene expression by transforming growth factor-beta 1. Evidence for two distinct mechanisms of inhibition at the transcriptional and posttranscriptional levels. J. Clin. Invest. 86, 1489–1495 (1990). 2122979 10.1172/JCI114866 PMC296894

[b43] Irintchev A., Zweyer M., Cooper R. N., Butler-Browne G. S. & Wernig A. Contractile properties, structure and fiber phenotype of intact and regenerating slow-twitch muscles of mice treated with cyclosporin A. Cell Tissue Res. 308, 143–156 (2002). 12012214 10.1007/s00441-002-0519-x

[b44] Miyabara E. H. . Thyroid hormone receptor-beta-selective agonist GC-24 spares skeletal muscle type I to II fiber shift. Cell Tissue Res. 321, 233–241 (2005). 15947969 10.1007/s00441-005-1119-3

